# Factors associated with implementation of the 5A’s smoking cessation model

**DOI:** 10.1186/s12971-017-0146-7

**Published:** 2017-11-02

**Authors:** C. Martínez, Y. Castellano, A. Andrés, M. Fu, L. Antón, M. Ballbè, P. Fernández, S. Cabrera, A. Riccobene, E. Gavilan, A. Feliu, A. Baena, M. Margalef, E. Fernández

**Affiliations:** 1 Tobacco Control Unit, Cancer Control and Prevention Programme, Institut Català d’Oncologia-ICO, Av. Granvia de L’Hospitalet, 199-203, E-08908 L’Hospitalet de Llobregat, Barcelona, Spain; 2 Cancer Control and Prevention Group, Institut d’Investigació Biomèdica de Bellvitge-IDIBELL, Av. Granvia de L’Hospitalet 199-203, 08908 L’Hospitalet de Llobregat, Barcelona, Spain; 3 Medicine and Health Sciences School, C. Josep Trueta s/n, 08915 Sant Cugat del Valles, Barcelona, Spain; 4 Department of Clinical Sciences, School of Medicine, Universitat de Barcelona, C. Feixa llarga s/n, 08907 L’Hospitalet del Llobregat, Barcelona, Spain; 5 National Institute of Physical Education of Catalonia (INEFC), Av. de l’Estadi, 12-22, 08038 Barcelona, Spain; 6 Addictions Unit, Institute of Neurosciences, Hospital Clínic de Barcelona, C. Villarroel 170, 08036 Barcelona, Spain; 7 Nursing Research Unit, Institut Català d’Oncologia-ICO, Av. Granvia de L’Hospitalet 199-203, 08908 L’Hospitalet de Llobregat, Barcelona, Spain

**Keywords:** Smoking cessation, Healthcare workers, Health organizations, Barriers, Facilitators

## Abstract

**Background:**

Several health organizations have adopted the 5A’s brief intervention model (Ask, Advise, Assess, Assist, Arrange), based on evidence-based guidelines for smoking cessation. We examine individual, cognitive, behavioral, and organizational factors associated with the 5A’s performance among clinical healthcare workers in Catalonia. We also investigate how these factors interact and potentially predict the implementation of each component of the 5A’s.

**Methods:**

A cross-sectional survey was conducted among clinical health workers enrolled in an online smoking cessation training course (*n* = 580). The survey included questions about individual characteristics as well as cognitive, behavioral, and organizational factors previously identified in research. We assessed self-reported performance of the 5A’s, assessed on a scale from 0 to 10, and used Multivariate regression to examine factors associated with its performance.

**Results:**

The performance means (standard deviation) were moderate for the first 3A’s [Ask: 6.4 (3.1); Advise: 7.1 (2.7); Assess: 6.3 (2.8)] and low for the last 2A’s [Assist: 4.4 (2.9); Arrange: 3.2 (3.3)]. We observed a high correlation between Assist and Arrange (*r* = 0.704, *p* < 0.001). Having positive experiences and feeling competent were positively associated with performing the 5A’s model and having organizational support with Assist and Arrange. Personal tobacco use among healthcare workers was negatively associated with Advice and Arrange.

**Conclusions:**

Our study found that clinical healthcare workers do not perform the 5A’s completely. The main barriers identified suggest the need of training and making available practical guidelines in healthcare services. Organizational support is essential for moving towards the implementation of Assist and Arrange.

**Electronic supplementary material:**

The online version of this article (10.1186/s12971-017-0146-7) contains supplementary material, which is available to authorized users.

## Background

Tobacco use is the leading cause of mortality worldwide, with tobacco-related diseases being responsible for 6 million deaths annually [1]. In 2005, the World Health Organization-Framework Convention on Tobacco Control (WHO-FCTC) promoted several policies to tackle the tobacco epidemic, including Article 14, which directs countries to implement smoking cessation services and calls on healthcare workers (HWs) and organizations to promote smoking cessation and offer support to tobacco users [2, 3].

Smokers are frequent users of health care services, and their contact with the health care system may facilitate quitting [4]. Hospitalization has been identified as a “teachable moment” for many smokers [5], and between 60% and 70% of patients make an attempt to quit while they are hospitalized [6]. Thus, hospitalization provides a unique opportunity to identify and engage smokers, initiate cessation treatments, and facilitate appropriate follow-up and support [7].

Internationally, several health organizations have adopted the 5A’s brief smoking cessation intervention model proposed by evidence-based guidelines for smoking cessation [8, 9]. This model is based on five strategies: (1) Ask patients about smoking at every visit, (2) Advise all tobacco users to quit, (3) Assess smokers’ willingness to try to quit, (4) Assist smokers’ efforts with treatment and referrals, and (5) Arrange follow-up contacts to support cessation efforts [8, 9]. However, deficiencies persist in implementing smoking cessation interventions in hospital settings as part of their routine practice [10, 11].

Earlier findings in health service and implementation research provided some understanding of some of the elements that facilitate and/or hamper smoking cessation implementation, including individual factors (e.g., health profession, smoking status); psychological and cognitive factors, such as motivation, knowledge, and confidence in providing the intervention [12–15], including negative or positive beliefs about smoking cessation interventions (e.g., time-consuming, ineffective, interfering with the professional-patient relationship, considered intrusive regarding the patient’s privacy) [16–18]; organizational factors, including organizational characteristics such as type of organization (public versus private), type of health care center (hospital, primary care center, etc.), and resources that may affect the practice (e.g., available time, access to protocols, records, educational materials, and pharmacological aids) [19–22]; and social support from supervisors, coworkers, and the organization [23–25].

Given that many health organizations have adopted the 5A’s intervention model, some studies have specifically focused on exploring performance factors for the components of this model. This research has indicated that being familiar with the National tobacco cessation guidelines (i.e. Public Health Services guidelines in the US), having had training, and believing that treatment is an important professional responsibility, are associated with high performance [26–28]. In contrast, the (healthcare workers) HWs factors such as being a smoker, not being a care physician, being uncomfortable asking patients if they smoke, believing counseling is not an appropriate service, and reporting competing priorities, are associated with low performance of the 5’As [26]. Although these factors have been identified, there is relatively little knowledge on how they interact in the performance of each of the As (Ask, Advice, Assess, Assist, and Arrange) as routine practice in health care organizations.

Implementation research has made it clear that exploring both individual and contextual factors is essential. Thus, there is increasing interest among implementation researchers in using theories concerning the organizational level given that the “setting” or “context” in which implementation occurs has been increasingly acknowledge as having an important influence in outcomes [29, 30]. Theories concerning organizational culture, organizational climate, leadership, and organizational learning are relevant for understanding and explaining organizational influences on the implementation process [29]. Therefore, many theoretical frameworks that are currently employed in implementation research are multilevel, identifying determinants at different levels, from the individual to the organization level [30]. These integrative frameworks recognize that implementation is a multidimensional phenomenon with multiple influences [29]. However, though some frameworks utilize a systems approach, determinants are often assessed individually assuming a linear relationship between determinants and the outcomes, ignoring that individual enablers and barriers may interact in various ways that can be difficult to predict [29].

In the present study, we explore several domains previously identified in research to assess the implementation of smoking cessation interventions among HWs from organizations belonging to the Catalan Network of Smoke Free Hospitals (Spain). This public health project has developed a scale-up tobacco control policy implementation model for healthcare organizations based on the Diffusion of Innovations theory [31].

Thus, the aims of the study were to describe smoking cessation practices according to the 5A’s model based on individual, cognitive, behavioral, and organizational factors among HWs in Catalonia (Spain), and to identify how these factors interact and potentially predict the implementation of each component of the 5A’s smoking cessation model. We hypothesize that smoking cessation practices are not broadly implemented, and Assist and Arrange in particular are infrequently applied. In addition, we expect that acceptable implementation of the 5A’s is associated with both cognitive and organizational factors.

## Methods

### Participants

HWs who enrolled in a brief online smoking cessation training course were approached to participate in the present cross-sectional study. This was a 6-h course created by the Catalan Network of Smoke Free Hospitals for HWs who work in member organizations (mainly hospitals). Recruitment was conducted through the course registration process immediately before starting the training. The survey was available via hyperlink and was a compulsory step for accessing the training course. A total of 715 HWs completed the online survey between January 2014 and March 2016. For duplicated entries, the most complete set of answers was used. Thirteen of the respondents left more than 20% of the questionnaire blank. Therefore, 699 participants (97.8%) completed the survey and provided the data reported here.

### Survey

The survey was a 63-item online questionnaire based on an instrument developed by Sheffer [32] to measure cognitive, and some behavioral, factors: 1) motivation, 2) knowledge about tobacco cessation, 3) self-efficacy, 4) importance of providing tobacco use interventions, 5) effectiveness of interventions, 6) importance of barriers, and 7) preparedness [32]. The survey also assessed the self-reported level of implementation of each of the components of the 5A’s model and included questions about responders’ individual characteristics, including sex, professional group (doctors, registered nurses, nurse assistants, others), tobacco use history, previous tobacco cessation education, and characteristics of their organization (e.g., public or private, hospital or other type of health care center). Finally, we included questions to explore the clinical environment and organizational-level characteristics identified previously in the literature and suggested by a panel of experts: having records, systematic protocols, access to tobacco cessation pharmacological aids, using additional resources to intervene, among others (Cronbach’s α=0.77) [12, 20, 21]. All 63 items were assessed on a scale of 0–10, with 0 being “none or not at all” and 10 being “the most possible”.

### Ethical considerations

All participants were informed about the main objectives of the study and provided informed consent for their voluntary participation. This study protocol was approved by the Ethics Committee of the Hospital Universitari de Bellvitge (PR040/15).

### Data analysis

Chi square analysis was used to compare HWs characteristics (sex, age, smoking status, years of working experience, previous training, type of center -hospital or another type of health care organization-, type of health administration –public or private-) by professional groups. Performance of the 5A’s intervention and knowledge of tobacco cessation resources were described by individual and organizational characteristics. The results were presented as mean and standard deviation (SD). To explore barriers and opportunities for the performance of each of the 5A’s, they were divided into two groups: acceptable performance (≥ 5) and not acceptable performance (< 5). This criterion was based on the results of Sheffer et al. study -developers of the questionnaire we used- (in their result the performance for Ask, Advise and Assess, scored >5, but Assist and Arrange ≤5) [32]. The significance of differences was assessed using Mann-Whitney U test and Kruskal-Wallis test.

Multivariate linear regression analysis was performed for each of the 5A’s. The variables related to barriers and opportunities for their implementation that revealed correlations with the outcome measure >0.30 were included in the analysis. For the linear regression analysis, selected individual characteristics were entered as fixed variables, and selected items related to barriers and opportunities were entered by stepwise method.

The criterion for significance was *p* < 0.05. Analyses were performed in SPSS version 21.

## Results

### Participant characteristics

Among all the participants (*n* = 699), we selected for these study those who reported developing clinical practice as their main responsibility (*n* = 580, 82% of them) (Table 1). Clinical HWs were similar to non-clinical ones in all the explored independent variables except for professional group, where other professional group was composed by mainly non-clinical workers (Table 1). Table 2 describes the characteristics of the sample for this study. The majority of the participants were women, never smokers, belonged to hospitals, and had previous training in smoking cessation. By professions we observe that Nursing Assistants were mainly >40 years/old, with >14 years of working experience, had higher smoking rates (30.5) compared to the rest of HWs, and 90.1% of them reported having receiving previous training in smoking cessation (Table 2).

**Table 1:** Characteristics of the clinical and non-clinicial participants by independent variables

	Overall		Clinical Health care workers		Non-clinical Health care workers		*p*
	n	%	n	%	n	%	
Overall	699	100	580	82.1	119	17.9	<0.001
Sex^a^							
Men	135	19.3	107	18.4	28	23.5	0.162
Women	559	80.0	471	81.2	88	73.9	
Age^a^							
< 40 years/old	342	48.9	291	50.2	51	42.9	0.199
≥ 40 years/old	305	43.6	248	42.8	57	47.9	
Professional group							
Doctor	52	7.4	43	7.4	9	7.6	<0.001
Registered nurses	375	53.6	345	59.5	30	25.2	
Nursing Assistants	143	20.5	131	22.6	12	10.1	
Others	129	18.5	61	10.5	68	57.1	
Working experience^a^							
< 14 years	326	46.6	271	46.7	55	46.2	0.756
≥ 14 years	326	46.6	268	46.2	58	48.7	
Smoking							
Smokers	170	24.3	137	23.6	33	27.7	0.603
Former smokers	213	30.5	177	30.5	36	30.3	
Never smokers	316	45.2	266	45,9	50	42.0	
Health care organization							
Hospital	592	84.7	488	84.1	104	87.4	0.369
Other health care organizations	107	15.3	92	15.9	15	12.6	
Previous Training							
No	565	80.8	469	80.9	96	80.7	0.962
Yes	134	19.2	111	19.1	23	19.3	

^a^Missings

**Table 2:** Characteristics of the sample by independent variables

	Overall		Doctors		Registered nurses		Nursing Assistants		Others		*p*
	n	%	n	%	n	%	n	%	n	%	
Overall	580	100	43	7.4	345	59.5	131	22.6	61	10.5	<0.001
Sex											
Men	107	18.5	23	54.8	43	12.5	20	15.3	21	34.4	<0.001
Women	471	81.5	19	45.2	301	87.5	111	84.7	40	65.6	
Age^a^											
< 40 years/old	291	54.0	23	54.8	199	62.2	38	32.2	31	52.5	<0.001
≥ 40 years/old	248	46.0	19	45.2	121	37.8	80	67.8	28	47.5	
Working experience											
< 14 years	271	50.3	24	55.8	168	51.7	52	44.8	27	49.1	0.533
≥ 14 years	268	49.7	19	44.2	157	48.3	64	55.2	28	50.9	
Smoking											
Smokers	137	23.6	5	11.6	72	20.9	40	30.5	20	32.8	<0.001
Former smokers	177	30.5	15	34.9	90	26.1	51	39.9	21	34.4	
Never smokers	266	45.9	23	53.5	183	53.0	40	30.5	20	32.8	
Health care organization											
Hospital	488	84.1	34	79.1	299	86.7	113	86.3	42	68.9	0.004
Other health care organizations	92	15.9	9	20.9	46	13.3	18	13.7	19	31.1	
Previous Training											
No	111	19.1	6	14.0	85	24.6	13	9.9	7	11.5	0.001
Yes	469	80.9	37	86.0	260	75.4	118	90.1	54	88.5	

^a^Missings

### Self-reported performance and knowledge of tobacco cessation resources

As shown in Table 3, HWs reported a higher average performance in Ask, Advise, and Assess than in Assist and Arrange. Doctors reported significantly higher scores for 5A’s performance than nurses and other HWs, except for Assist (Table 3). In addition, non-smokers and previously trained HWs reported significantly higher performance of each of the 5A’s than smokers and non-trained HWs, respectively. No differences were found according to years of experience and age groups.

**Table 3:** Self-reported performance of the 5A’s intervention, development of some related activities and knowledge of tobacco cessation resources by independent variables (individual and by organization)

	Ask		Advise		Assess		Assist		Arrange	
	mean (sd)	*p*	mean (sd)	*p*	mean (sd)	*p*	mean (sd)	*p*	mean (sd)	*p*
Overall	6.4 (3.1)		7.1 (2.7)		6.3 (2.8)		4.4 (2.9)		3.2 (3.3)	
Individual characteristics										
Profession										
Doctors	8.5 (2.4)	<.001	8.1 (2.7)	0.001	7.0 (2.5)	0.018	4.4 (2.9)	0.069	3.8 (3.2)	0.027
Nurses	7.0 (2.9)		7.2 (2.5)		6.5 (2.6)		4.6 (3.0)		3.4 (3.4)	
Others	4.9 (3.2)		6.7 (3.0)		5.8 (3.1)		4.0 (2.9)		2.7 (3.0)	
Sex										
Women	6.3 (3.1)	0.102	7.0 (2.7)	0.304	6.3 (2.8)	0.715	4.4 (2.9)	0.450	3.1 (3.3)	0.477
Men	6.8 (3.2)		7.2 (2.9)		6.2 (2.8)		4.6 (3.0)		3.4 (3.2)	
Smoking										
Smokers	5.8 (3.3)	0.006	6.1 (2.9)	<.001	5.9 (2.9)	0.046	3.9 (2.7)	0.018	2.5 (2.8)	0.014
Former smokers	6.4 (3.1)		7.5 (2.6)		6.5 (2.7)		4.7 (3.0)		3.3 (3.4)	
Never smokers	6.7 (3.0)		7.3 (2.6)		6.4 (2.7)		4.5 (3.0)		3.5 (3.3)	
Age										
< 40 years/old	6.5 (3.0)	0.568	6.8 (2.7)	0.001	6.1 (2.8)	0.049	4.3 (2.8)	0.378	2.9 (3.0)	0.226
≥ 40 years/old	6.3 (3.3)		7.4 (2.8)		6.5 (2.8)		4.5 (3.1)		3.4 (3.5)	
Working experience										
< 14 years	6.3 (3.0)	0.094	6.8 (2.7)	0.001	6.2 (2.7)	0.126	4.3 (2.8)	0.387	3.1 (3.1)	0.476
≥ 14 years	6.6 (3.2)		7.4 (2.7)		6.5 (2.7)		4.5 (3.0)		3.3 (3.4)	
Previous Training										
Yes	7.2 (2.5)	0.023	7.8 (2.2)	0.003	7.1 (2.4)	0.002	5.8 (2.8)	<.001	4.9 (3.3)	<.001
No	6.2 (3.3)		6.9 (2.8)		6.1 (2.8)		4.1 (2.9)		2.8 (3.1)	
Organizational characteristics										
Type of center										
Hospital	6.3 (3.1)	0.158	7.1 (2.8)	0.961	6.3 (2.8)	0.125	4.4 (3.0)	0.291	3.1 (3.2)	0.021
Others	6.8 (3.2)		7.1 (2.7)		6.7 (2.8)		4.7 (2.8)		3.9 (3.3)	
Type of organization										
Public	6.6 (3.1)	0.014	7.1 (2.7)	0.942	6.3 (2.7)	0.588	4.4 (2.9)	0.702	3.1 (3.2)	0.540
Private	5.8 (3.3)		7.0 (3.0)		6.4 (3.0)		4.5 (3.0)		3.4 (3.3)	

Based on organizational characteristics, the performance of each component was similar between type of center (public vs. private) and type of organization (hospital vs. other). However, HWs from health centers other than hospitals reported higher rates of follow-ups than workers from hospitals (Table 2).

### Correlations of the performance of the 5A’s smoking cessation components

The results of correlation analysis using the 5A’s have showed a significant positive pairwise correlation among each of the components (Additional file 1: Table S1). However, Ask, Advise, and Asses correlated well among them, and independently Assist and Arrange did between them. It seems that the higher the component of the 5A’s model, the better the performance of the next one. For example, Assist and Arrange [*r* = 0.704 (*p* = 0.01)], and Assist and Assess [*r* = 0.638 (*p* = 0.01)] present a high correlation but do not correlate well with Ask and Advice (2 first components of the model). This finding shows a different pattern of performance between those who explore smoking consumption by Asking, Advising and Assessing versus those who perform a genuine quitting aid by Assisting and Arranging.

### Barriers and opportunities for performance of the 5A’s model

Table 4 summarizes the most significant barriers and opportunities identified. Overall, HWs exhibited a high motivation to help patients quit, gave importance to smoking cessation in their jobs, and expressed an interest in receive further training. However, HWs reported a low level of preparedness overall and in the use of smoking cessation drugs, low familiarity with practical guidelines, low previous positive experiences helping to quit, and low perception of support by their organizations.

**Table 4:** Cognitive, behavioral, and organizational barriers and opportunities performing the 5A’s model

		Ask			Advise			Assess			Assist			Arrange		
	Overall	Acceptable (≥ 5)	Low (< 5)		Acceptable (≥ 5)	Low (< 5)		Acceptable (≥ 5)	Low (< 5)		Acceptable (≥ 5)	Low (< 5)		Acceptable (≥ 5)	Low (< 5)	
	mean (SD)	mean (SD)	mean (SD)	*p*	mean (SD)	mean (SD)	*p*	mean (SD)	mean (SD)	*p*	mean (SD)	mean (SD)	*p*	mean (SD)	mean (SD)	*p*
Cognitive Factors																
Motivation to help patients to quit	8.1 (1.9)	8.4 (1.6)	7.4 (2.5)	<.001	8.4 (1.6)	6.8 (2.5)	<.001	8.4 (1.6)	7.3 (2.5)	<.001	8.5 (1.5)	7.8 (2.1)	<.001	8.6 (1.4)	7.9 (2.0)	<.001
Importance of cessation in their job	8.2 (1.1)	8.5 (1.8)	7.4 (2.4)	<.001	8.5 (1.7)	6.9 (2.5)	<.001	8.5 (1.7)	7.2 (2.5)	<.001	8.6 (1.6)	7.8 (2.2)	<.001	8.7 (1.4)	8.0 (2.2)	0.001
Self-reported preparedness	4.4 (2.6)	4.8 (2.5)	3.2 (2.5)	<.001	4.7 (2.5)	2.1 (2.2)	<.001	4.7 (2.5)	3.2 (2.4)	<.001	5.6 (2.2)	3.2 (2.3)	<.001	5.9 (2.1)	3.6 (2.4)	<.001
Preparedness in using drugs	3.4 (2.7)	3.8 (2.8)	2.1 (2.2)	<.001	3.7 (2.8)	2.0 (2.1)	<.001	3.8 (2.7)	2.2 (2.3)	<.001	4.6 (2.6)	2.2 (2.3)	<.001	5.2 (2.5)	2.5 (2.3)	<.001
Competency in assisting smokers	5.0 (2.6)	5.5 (2.4)	3.2 (2.3)	<.001	5.4 (2.5)	3.0 (2.3)	<.001	5.4 (2.4)	3.2 (2.5)	<.001	6.1 (2.2)	3.7 (2.4)	<.001	6.5 (2.0)	4.2 (2.5)	<.001
Security to motivate smokers	6.3 (2.1)	6.7 (1.9)	5.0 (2.2)	<.001	6.7 (1.8)	4.5 (2.3)	<.001	6.7 (1.8)	4.9 (2.5)	<.001	7.1 (1.5)	5.5 (2.3)	<.001	7.2 (1.4)	5.8 (2.2)	<.001
Wish more training	8.3 (2.1)	8.5 (1.8)	7.6 (2.5)	<.001	8.6 (1.8)	7.1 (2.7)	<.001	8.6 (1.7)	7.3 (2.8)	<.001	8.6 (1.7)	8.0 (2.3)	0.003	8.6 (1.6)	8.1 (2.3)	0.043
It is part of my job	6.4 (3.3)	7.1 (2.9)	4.1 (3.3)	<.001	6.8 (3.1)	4.6 (3.5)	<.001	6.9 (2.1)	4.5 (3.6)	<.001	7.4 (2.7)	5.3 (3.5)	<.001	7.6 (2.6)	5.7 (3.4)	<.001
Behavioral Factors																
Uses additional resources to intervene	5.8 (2.5)	6.3 (2.2)	4.4 (2.9)	<.001	6.2 (2.3)	3.9 (3.0)	<.001	6.2 (2.2)	4.3 (3.1)	<.001	6.7 (2.0)	4.9 (2.8)	<.001	6.6 (1.9)	5.4 (2.8)	<.001
Frequency in seeing tobacco-related diseases	7.8 (2.1)	8.1 (1.8)	6.8 (2.5)	<.001	8.1 (1.8)	6.4 (2.6)	<.001	8.0 (1.8)	6.9 (2.6)	<.001	8.1 (1.7)	7.4 (2.4)	0.001	8.2 (1.5)	7.5 (2.3)	0.006
Familiar with practical guidelines for smoking cessation	2.4 (2.7)	2.9 (2.8)	0.8 (1.4)	<.001	2.7 (2.8)	0.7 (1.2)	<.001	2.8 (2.8)	0.8 (1.5)	<.001	3.6 (2.8)	1.1 (1.7)	<.001	4.6 (2.7)	1.2 (1.8)	<.001
Previous positive experiences	3.7 (3.2)	4.3 (3.1)	1.8 (2.4)	<.001	4.1 (3.1)	1.5 (2.3)	<.001	4.3 (3.1)	1.6 (2.2)	<.001	5.2 (2.1)	1.1 (2.4)	<.001	5.8 (2.8)	2.5 (2.7)	<.001
Organizational and Support Factors																
It is protocolized in my organization	4.5 (3.4)	4.9 (3.3)	2.9 (3.0)	<.001	4.8 (3.4)	2.8 (2.1)	<.001	4.9 (3.3)	3.0 (3.2)	<.001	5.6 (3.2)	3.2 (3.1)	<.001	6.0 (2.1)	3.6 (3.3)	<.001
Required by supervisors	3.1 (3.3)	4.5 (3.4)	2.4 (2.6)	<.001	4.3 (3.4)	2.3 (2.6)	<.001	4.4 (3.3)	2.5 (2.1)	<.001	5.2 (3.3)	2.7 (2.9)	<.001	5.6 (3.1)	3.1 (3.1)	<.001
Organizational support	4.7 (3.2)	5.2 (3.2)	3.0 (2.8)	<.001	5.0 (3.2)	2.1 (2.9)	<.001	5.1 (3.2)	3.1 (3.0)	<.001	5.1 (2.1)	3.3 (2.9)	<.001	6.6 (2.8)	3.7 (3.0)	<.001

To explore which of these factors could be considered barriers and/or opportunities, we compared the average means for HWs who acceptably implemented each component of the 5A’s intervention and those who did not. Results showed that those who acceptably implemented each of the As reported higher in all the cognitive factors explored (see Table 4) The cognitive factor that obtained higher difference between acceptable performers and low performers was considering smoking cessation as part of their job (with a difference of 3 points for Ask and 1.9 for Arrange). The behavioral factors that obtained less score were familiarity with practical guidelines and previous positive experiences, however as higher the 5A’s component of the model these factors were higher scored. All organizational factors were low scored, however in all the cases the higher the components of the 5A’s model the higher the scored obtained. Thus, those who acceptable performed Assist and Arrange reported having more organizational support by the three items explored (having smoking cessation protocolized in their organization, being required by their supervisors and perceiving organizational support).

### Factors associated with performance of each component of the 5A’s smoking cessation intervention model

Multiple linear regression analysis was used (Table 5) to develop a model to predict factors that increase HWs’ performance of each of the 5A’s, using factors with correlations >0.30 in the previous analysis. The final adjusted model for predicting Ask explained 42.5% of the variance in the implementation of this first component. One individual factor, being either a doctor or a nurse, was positively associated with the implementation of Ask. Several cognitive and behavioral factors were positively associated: considering having competency in assisting smokers, security to motivate smokers to quit, considering that smoking cessation is part of their job, using additional resources to intervene, seeing frequently patients with smoking-related diseases and having previous positive experiences in quit attempts. One organizational factor was also positively associated: cessation being required by their supervisors, explaining 10% of the variance alone, corresponding to 23.5% of the variability explained by the proposed model.

**Table 5:** Multivariate model predicting each of the components of the 5A’s model

Dependent variable	Ask ^adjusted^			Advise ^adjusted^			Assess ^adjusted^			Assist ^adjusted^			Arrange ^adjusted^		
Independent variable	Estimate	95% CI	*p*	Estimate	95% CI	*p*	Estimate	95% CI	p	Estimate	95% CI	*p*	Estimate	95% CI	*p*
Individual															
Smoking status (smoke)	−0.232	(−0.790; 0.327)	0.415	−0.630	(−1.099–0.162)	0.008	−0.027	(−0.543; 0.489)	0.918	−0.234	(−0.670; 0.202)	0.292	−0.771	(−1.306;-0.235)	0.005
Smoking status (former smoke)	−0.031	(−0.545; 0.484)	0.906	0.069	(−0.357; 0.496)	0.749	−0.019	(−0.495; 0.456)	0.936	0.190	(−0.207; 0.586)	0.348	−0.110	(−0.604; 0.384)	0.662
Sex (male)	0.197	(−0.369; 0.763)	0.495	0.049	(−0.426; 0.524)	0.841	−0.009	(−0.529; 0.512)	0.974	0.058	(−0.383; 0.499)	0.795	0.143	(−0.409; 0.695)	0.611
Previous education (yes)	−0.482	(−1.064; 0.099)	0.104	0.182	(−0.289; 0.654)	0.448	−0.259	(−0.787; 0.269)	0.336	−0.482	(−0.945;-0.019)	0.041	−0.074	(−0.626; 0.478)	0.792
Profession (doctor)	2.155	(1.253; 3.056)	0.000	0.304	(−0.447; 1.056)	0.427	0.138	(−0.702; 0.979)	0.747	−0.819	(−1.523;-0.115)	0.023	−0.181	(−1.042; 0.680)	0.679
Profession (nurse)	0.640	(0.100; 1.180)	0.020	−0.420	(−0.831;-0.009)	0.045	−0.353	(−0.848; 0.142)	0.162	−0.232	(−0.617; 0.152)	0.236	−0.184	(−0.658; 0.291)	0.448
Cognitive															
Motivation to help patients to quit	–			0.177	(0.048; 0.306)	0.007	–			–			–		
Importance of cessation in their job	–			0.130	(0.012; 0.249)	0.031	0.151	(0.029; 0.274)	0.015	–			–		
Self-reported preparedness	–			–			–			0.183	(0.093; 0.273)	<0.001	–		
Preparedness in using drugs	–			–			–			–			0.107	(0.001; 0.213)	0.048
Competency in assisting smokers	0.157	(0.050; 0.264)	0.004	–			0.126	(0.027; 0.224)	0.012	0.201	(0.117; 0.285)	<0.001	0.184	(0.082; 0.286)	<0.001
Security to motivate smokers	0.151	(0.019; 0.283)	0.025	0.183	(0.076; 0.291)	0.001	0.151	(0.029; 0.273)	0.016	–			–		
Wish more training	–			0.226	(0.125; 0.327)	<0.001	0.130	(0.019; 0.241)	0.022	–			–		
It is part of my job	0.236	(0.150; 0.323)	<0.001	–			0.111	(0.032; 0.189)	0.006	–			–		
Behavioral factors															
Uses additional resources to intervene	0.139	(0.043; 0.236)	0.005	0.146	(0.067; 0.226)	<0.001	0.191	(0.104; 0.278)	<0.001	0.202	(0.132; 0.272)	<0.001	–		
Frequency in seeing tobacco-related diseases	0.221	(0.103; 0.340)	<0.001	0.277	(0.177; 0.376)	<0.001	–			–			–		
Familiar with practical guidelines for smoking cessation	–			–			–			0.188	(0.106; 0.269)	<0.001	0.423	(0.317; 0.529)	<0.001
Previous positive experiences	0.099	(0.013; 0.185)	0.024	0.130	(0.067; 0.193)	<0.001	0.164	(0.086; 0.242)	<0.001	0.242	(0.172; 0.312)	<0.001	0.198	(0.114; 0.282)	<0.001
Organizational and Support Factors															
It is protocolized in my organization	–			–			0.091	(0.024; 0.158)	0.008	0.070	(0.003; 0.136)	0.042	–		
Required by supervisors	0.080	(0.003; 0.158)	0.043	–			–			–			–		
Organizational support	–			–			–			0.085	(0.013; 0.158)	0.021	0.161	(0.086; 0.236)	<0.001
R^2^	0.425			0.459			0.394			0.582			0.523		

Adjusted for sociodemographic variables (sex, age, professional group, smoking consumption, years of experience)

The adjusted model for predicting Advice explained 45.9% of its variance. Several cognitive and factors were positively associated: being motivated to help patients to quit, giving importance of smoking cessation in their job, reporting security to motivate smokers to quit, and reporting interest in receiving further training. Among the behavioral factors: using additional resources, having positive experience and frequently seeing tobacco-related diseases. On the other hand, being a smoker was significantly negatively associated with the performance of Advice (Table 5).

The adjusted model for Assess predicted 39.4% of the variance. Several cognitive and behavioral factors predicted the performance of Assess: giving importance of smoking cessation in their job, reporting having competency in assisting smokers, security in motivating smokers, wishing more training, and claiming that it is part of their job. In addition, one organizational factor predicted the implementation of Assess (being protocolized in their organization), explaining 9.7% of the variance alone, corresponding to 24.6% of the variability explained by the proposed model (Table 5).

The adjusted model for Assist predicted 58.2% of the variance. Several factors were positively associated with Assist, including two cognitive factors (perceiving preparedness and competency in assisting smokers), three behavioral factors (using additional resources, being familiar with practical guidelines and having previous positive experiences), and two organizational support-related factors (being protocolized in their organization and having organizational support). Organizational support alone explained 25.6% of the variance, corresponding to 44.0% of the variability explained in this model. Two factors were negatively associated with Assist: having previous training in smoking cessation and being a doctor compared to other professionals.

Finally, the Arrange model predicted 52.3% of the performance of this component. Being a HW tobacco user was negatively associated with preforming Arrange. Whereas, factors that seemed to increase Arranging a follow up were: two cognitive (having preparedness in the use of drugs, reporting competency in assisting smokers to quit) two behavioural (being familiar with practical guidelines and having previous positive experiences) and one organizational factor (having organizational support). Organizational support alone explained 21.7% of the variability, corresponding to 41.5% of the variability in the model.

In these models, we observed that having positive experiences was the most recurrent factor for the performance of each of the components of the 5A’s model.

## Discussion

In this study, HWs reported a higher level of performance in Ask, Advice, and Assess, but much lower in Assist and Arrange when approaching smokers about quitting. In addition, we observed a different pattern of performance between the first components and the last two, as shown in the correlation analysis. The multivariate analysis showed that one behavioural factor is positively associated with the 5A’s delivery: having positive experiences. For the performance of the Assist and Arrange, an organizational factor was necessary: having organizational support. Being a smoker was negatively associated with Advice and Arrange, but not for the other components.

The low level of performance in 5A’s delivery, in particular Assist and Arrange is consistent with previous research. Similar to our study, but providing the results in proportions, U.S. health professionals [26] reported that 87.3%–99.5% of HWs Ask patients to quit, 65.6%–94.9% offer Advice, and much fewer Assess the smoker’s interest to quit (38.7%–84.8%), Assist them (16.4%–63.7%), and Arrange a follow-up (1.3%–23.1%). This finding is also consistent with previous research [14, 33] pointing out how the application of the 5A’s in health organizations (in our case mainly acute hospitals) is mostly limited to brief interventions [34], such as the first three As (Ask, Advice, Assess). This confirms that intensive intervention practices recommended by guidelines are unlikely to be fully implemented in some health care settings. Some researchers/clinicians have suggested a briefer model for hospital settings [34–36]. There has also been some debate about the scope of cessation counseling in hospitals, with some experts advocating a shorter model (“Ask-Advise-Refer/Act”) [34–36].

Our study only used HWs’ self-reported information; so, we assume a low level of recorded smoking cessation activity in Catalan health care services (mainly hospitals) could be found.

Our findings are important to understand the factors that increase HW involvement in the implementation of each of the components of the 5A’s. Linear regression analysis revealed several factors that were significantly associated with their implementation, explaining 44.2% to 61.6% of the smoking cessation practices. The main factors involved in smoking cessation interventions were individual, cognitive, behavioral and organizational.

The main individual barriers encountered were being a smoker (versus being a former and/or never smoker) and being neither a doctor nor a nurse, as identified in previous studies [26, 33]. However compared to previously reported, in our study, being a smoker was negatively associated with performance in Advice and Arrange. In addition, doctors reported higher performance in each of the components compared to other health professional groups [26, 37].

Regarding psychological and cognitive factors, we identified having competence and experience as important facilitators for performing each of the components of the 5A’s model. In addition, HWs who performed Ask, Advice and Assess believed that providing support for cessation is part of their job, but this relationship was not found for Assist and Arrange. Our study also found that the lack of self-reported preparedness and the unfamiliarity with practical guidelines were important barriers in performing the 5A’s. In a systematic review, the most common negative beliefs that hamper smoking cessation were: time-consuming, ineffective, lack of confidence in their ability to discuss smoking with their patients, unpleasant feeling in discussing smoking consumption with patients, and lack of confidence in their knowledge [16]. We included these factors in our study, but the multivariate analysis did not identify a clear barrier, but only potential opportunities (competency in assisting smokers, using additional resources and previous positive experiences). This finding should be taken into account in the development and implementation of future smoking cessation programs.

A strong relationship has been recognized between training and increased levels of confidence, higher performance, and fewer reported barriers to provide cessation services [14]. However, in our study, we did not perform this analysis, but observed that higher levels of confidence are related to higher performance of the 5A’s. HWs who received previous training scored with a higher performance for each of the components (see Table 2) from those without training. The averaged mean was approximately 2-fold higher for the activities Assisting, Arranging, when HWs had received training. However, in our multivariate linear regression model, previous training became a less significant predictor. This finding could indicate the need for training, especially among nurses and other health professionals. Online training using the 5A’s model is effective for improving knowledge and self-confidence in smoking cessation skills [38, 39], but this needs to integrate simulation as an effective way to assist students in the acquisition of communication and performance skills [39, 40].

Finally, and consistent with previous findings of the importance of the organizational level, we found that receiving organizational support increases the 5A’s delivery [23–25]. However, we identified having organizational support as the most vital factor for moving forward to Assist and Arrange. In a previous study, most of the variance in the model implementation occurred at the counselor level, the unconditional means model showed that nearly one-quarter of the variance was attributable to differences between organizations [41]. In our study, we found that perceived organizational support explains between 44.5% and 41.5% of the variance of Assist and Arrange, respectively.

Organizational leadership and support in adopting the innovation, as well as the compatibility between an innovation and the beliefs of the individuals who are responsible for implementation, are key to its correct application [31, 42]. If individuals perceive that the organizational context contains a variety of barriers to use an innovation, they are less likely to implement it [43]. This could be a possible explanation for understanding why training does not always increase the frequency of interventions [14, 32]; for example, they interact with organizational and contextual factors that could rule out the implementation of smoking cessation interventions.

### Limitations

Several limitations should be noted for this study. First, this is a cross-sectional survey and we are not able to reach direct causal conclusions about our results. Second, this study relies on self-reported responses. HWs’ smoking cessation practices and smoking status were not verified, nor information about organizational factors. Third, our participants were HWs enrolled in smoking cessation training and may not be representative of the general characteristics of the HWs in Catalonia. Fourth, due to the convenient nature of our sample, we could have introduced compliance bias, as our participants could have more interest in smoking cessation practices and provide more positive responses. However, this study explores, for the first time in Spain, several individual, psychological, and organizational factors related to the implementation of the 5A’s model. Finally, we included 580 HWs with primary clinical responsibilities to avoid biased data in the performance of the 5A’s model due to the nature of their professional activity and then increase the internal and external validity of our results.

## Conclusions

This study adds to the literature on the implementation of smoking cessation interventions, highlighting the importance of organizational context, as well as individual beliefs in the implementation process. In this study, we investigated the interaction between individual, cognitive, behavioral, and organizational factors and 5A’s smoking cessation performance. Although a previous study also searched for this interaction [25], it did not investigate the interaction of each of the components of the 5A’s model. Our results suggest some practical implications, such as facilitating practical training that includes cases, examples, workshops, and sessions in which professionals can build their practical skills; training and involving HWs, especially nurses and other health professionals, in smoking cessation; providing managerial and organizational support, such as promoting professionals who are active in smoking cessation; and facilitating resources (tobacco cessation drugs, materials, etc.) and continuous and specialized smoking cessation training.

In conclusion, the present study shows that the 5A’s model is not fully implemented among Catalan HWs, with an important difference between the implementation of Ask, Advise, Assess and Assist and Arrange. The common facilitators for performing the 5A’s were having positive experiences and feeling competent in helping others to quit. However, in order strengthen Assist and Arrange gaining confidence seems necessary. This could be gained through practical workshops in which HWs could learn about the usage of available drugs, and make familiar with community smoking cessation resources (e.g., quit lines, printed materials, websites, etc). In addition, perceiving organizational support is vital for improving the level of 5A’s implementation. These facilitators should be taken into account to improve smoking cessation intervention implementation in health care settings.

## Additional file

Additional file 1: Table S1. Correlations between each of the components of the 5A’s. (DOCX 13 kb)

## Abbreviations

HW: Healthcare workers
WHO-FCTC: World Health Organization-Framework Convention on Tobacco Control

## References

[1] World Health Organization. WHO report on the global tobacco epidemic. 2013.

[2] World Health Organization, ed. FCTC/COP4(8): Guidelines for implementation of article 14 of the WHO framework convention on tobacco Control. http://www.who.int/fctc/Guidelines.pdf?ua=1.; 2010. Accessed October 18, 2016.

[3] McRobbie H, Raw M, Chan S (2013). Research priorities for article 14--demand reduction measures concerning tobacco dependence and cessation. Nicotine Tob Res.

[4] Duffy SA, Scholten RL, Karvonen-Gutierrez CA (2010). The relation of tobacco use during hospitalization to post-discharge smoking cessation among US veterans. Prev Med.

[5] McBride CM, Ostroff JS (2003). Teachable moments for promoting smoking cessation: the context of cancer care and survivorship. Cancer Control.

[6] Zack E (2002). Smoking withdrawal and prolonged hospitalization. Clin J Oncol Nurs.

[7] Rigotti NA, Clair C, Munafo MR, Stead LF (2012). Interventions for smoking cessation in hospitalised patients. Cochrane Database Syst Rev.

[8] Fiore MC, Baker TB (2011). Clinical practice. Treating smokers in the health care setting. N Engl J Med.

[9] Five major steps to intervention (the "5 A's"). December 2012. Agency for healthcare research and quality, rockville, MD. http://www.ahrq.gov/professionals/clinicians-providers/guidelines-recommendations/tobacco/5steps.html. Accessed October 11, 2016.

[10] Freund M, Campbell E, Paul C (2008). Smoking care provision in hospitals: a review of prevalence. Nicotine Tob Res.

[11] Martinez C, Fu M, Martinez-Sanchez JM (2009). Tobacco control policies in hospitals before and after the implementation of a national smoking ban in Catalonia, Spain. BMC Public Health.

[12] Sarna L, Bialous SA, Wells M, Kotlerman J, Wewers ME, Froelicher ES (2009). Frequency of nurses' smoking cessation interventions: report from a national survey. J Clin Nurs.

[13] Sarna L, Wewers ME, Brown JK, Lillington L, Brecht ML (2001). Barriers to tobacco cessation in clinical practice: report from a national survey of oncology nurses. Nurs Outlook.

[14] Applegate BW, Sheffer CE, Crews KM, Payne TJ, Smith PO (2008). J Eval Clin Pract.

[15] Smit ES, de Vries H, Hoving C (2013). Determinants of practice nurses' intention to implement a new smoking cessation intervention: the importance of attitude and innovation characteristics. J Adv Nurs.

[16] Vogt F, Hall S, Marteau TM (2005). General practitioners' and family physicians' negative beliefs and attitudes towards discussing smoking cessation with patients: a systematic review. Addiction.

[17] Hall S, Marteau TM (2007). Practice nurses' self-reported opportunistic smoking cessation advice in three contexts. Nicotine Tob Res.

[18] Godin G, Belanger-Gravel A, Eccles M, Grimshaw J (2008). Healthcare professionals' intentions and behaviours: A systematic review of studies based on social cognitive theories. Implement Sci.

[19] Smith PM, Sellick SM, Spadoni MM (2012). Tobacco cessation clinical practice guideline use by rural and urban hospital nurses: A pre-implementation needs assessment. BMC Nurs.

[20] Leitlein L, Smit ES, de Vries H, Hoving C (2012). Factors influencing dutch practice nurses' intention to adopt a new smoking cessation intervention. J Adv Nurs.

[21] Freund M, Campbell E, Paul C (2009). Increasing smoking cessation care provision in hospitals: a meta-analysis of intervention effect. Nicotine Tob Res.

[22] Eby LT, Laschober TC, Muilenburg JL (2014). Understanding counselors' implementation of tobacco cessation services with patients. J Subst Abus Treat.

[23] Segaar D, Bolman C, Willemsen MC, Vries H (2006). Determinants of adoption of cognitive behavioral interventions in a hospital setting: example of a minimal-contact smoking cessation intervention for cardiology wards. Patient Educ Couns.

[24] Choi SH, Kim YH (2016). Factors affecting Korean registered nurses' intention to implement smoking cessation intervention. Osong Public Health Res Perspect.

[25] Laschober TC, Muilenburg JL, Eby LT (2015). Factors linked to substance use disorder counselors' (non)implementation likelihood of tobacco cessation 5 A's, counseling, and pharmacotherapy. J Addict Behav Ther Rehabil.

[26] Tong EK, Strouse R, Hall J, Kovac M, Schroeder SA. National survey of U.S. health professionals' smoking prevalence, cessation practices, and beliefs. Nicotine Tob Res. 2010; doi:10.1093/ntr/ntq071. 10.1093/ntr/ntq071 PMC6281036 20507899

[27] Papadakis S, Gharib M, Hambleton J, Reid RD, Assi R, Pipe AL (2014). Delivering evidence-based smoking cessation treatment in primary care practice: experience of Ontario family health teams. Can Fam Physician.

[28] Papadakis S, Cole AG, Reid RD, Coja M, Aitken D, Mullen KA, Gharib M, Pipe AL (2016). Increasing rates of tobacco treatment delivery in primary care practice: evaluation of the Ottawa model for smoking cessation. Ann Fam Med.

[29] Nilsen P (2015). Making sense of implementation theories, models and frameworks. Implement Sci.

[30] Michie S, van Stralen MM, West R. The behaviour change wheel: A new method for characterising and designing behaviour change interventions. Implement Sci. 2011;6:42. doi:10.1186/1748-5908-6-42. 10.1186/1748-5908-6-42 PMC3096582 21513547

[31] Rogers EM (2003). Diffusion of innovations.

[32] Sheffer CE, Barone CP, Anders ME (2009). Training health care providers in the treatment of tobacco use and dependence: pre- and post-training results. J Eval Clin Pract.

[33] Tremblay M, Cournoyer D, O'Loughlin J (2009). Do the correlates of smoking cessation counseling differ across health professional groups?. Nicotine Tob Res.

[34] Berndt NC, Bolman C, de Vries H, Segaar D, van Boven I, Lechner L (2013). Smoking cessation treatment practices: recommendations for improved adoption on cardiology wards. J Cardiovasc Nurs.

[35] Schroeder SA (2012). Smoking among hospitalized patients: another opportunity to improve patients' health. Arch Intern Med.

[36] Reid RD, Mullen KA, Slovinec D'Angelo ME (2010). Smoking cessation for hospitalized smokers: an evaluation of the "Ottawa model". Nicotine Tob Res.

[37] Meshefedjian GA, Gervais A, Tremblay M, Villeneuve D, O'Loughlin J (2010). Physician smoking status may influence cessation counseling practices. Can J Public Health.

[38] Schmelz AN, Nixon B, McDaniel A, Hudmon KS, Zillich AJ (2010). Evaluation of an online tobacco cessation course for health professions students. Am J Pharm Educ.

[39] Shishani K, Stevens K, Dotson J, Riebe C (2013). Improving nursing students' knowledge using online education and simulation to help smokers quit. Nurse Educ Today.

[40] Nestel D, Tierney T (2007). Role-play for medical students learning about communication: guidelines for maximising benefits. BMC Med Educ.

[41] Knudsen HK, Studts CR, Studts JL (2012). The implementation of smoking cessation counseling in substance abuse treatment. J Behav Health Serv Res.

[42] Fixsen DL, Naoom SF, Blase KA, Friedman RM, Wallace F (2005). Implementation Research: A Synthesis of the Literature. The National Implementation Research Network: Tampa.

[43] Klein KJ, Conn AB, Sorra JS (2001). Implementing computerized technology: an organizational analysis. J Appl Psychol.

