# Supplementary material for: Factors associated with implementation of the 5A’s smoking cessation model
Source: Tob Induc Dis. 2017 Nov 2;15:41. doi: 10.1186/s12971-017-0146-7 (PMC5669025; doi:10.1186/s12971-017-0146-7)
Supplement: Additional file 1: Table S1. — Correlations between each of the components of the 5A’s. (DOCX 13 kb) [file 12971_2017_146_MOESM1_ESM.docx]

**Additional file 1: Table S1. Correlations between each of the components of the 5A’s**

|  | | **Ask** | **Advise** | **Assess** | **Assist** | **Arrange** |
| --- | --- | --- | --- | --- | --- | --- |
| **Ask** | R | 1 |  |  |  |  |
|  | P |  |  |  |  |  |
|  | N | 579 |  |  |  |  |
| **Advise** | R | ,585** | 1 |  |  |  |
|  | p | ,000 |  |  |  |  |
|  | N | 578 | 579 |  |  |  |
| **Assess** | R | ,578** | ,650** | 1 |  |  |
|  | p | ,000 | ,000 |  |  |  |
|  | N | 576 | 576 | 577 |  |  |
| **Assist** | R | ,500** | ,486** | ,638** | 1 |  |
|  | p | ,000 | ,000 | ,000 |  |  |
|  | N | 579 | 579 | 577 | 580 |  |
| **Arrange** | R | ,406** | ,385** | ,482** | ,704** | 1 |
|  | p | ,000 | ,000 | ,000 | ,000 |  |
|  | N | 579 | 579 | 577 | 580 | 580 |
| **. Significance at 0,01 bilateral.  R: Pearson correlación coeficiente | | | | | | |
